# Characterization of a novel *LmSAP* gene promoter from *Lobularia maritima*: Tissue specificity and environmental stress responsiveness

**DOI:** 10.1371/journal.pone.0236943

**Published:** 2020-07-31

**Authors:** Rania Ben Saad, Walid Ben Romdhane, Nabil Zouari, Anis Ben Hsouna, Marwa Harbaoui, Faical Brini, Thaura Ghneim-Herrera

**Affiliations:** 1 Biotechnology and Plant Improvement Laboratory, Centre of Biotechnology of Sfax, University of Sfax, Sfax, Tunisia; 2 Plant Production Department, College of Food and Agricultural Sciences, King Saud University, Riyadh, Saudi Arabia; 3 Departments of Life Sciences, Faculty of Sciences of Gafsa, Gafsa, Tunisia; 4 Departamento de Ciencias Biológicas, Universidad Icesi, Cali, Colombia; National Taiwan University, TAIWAN

## Abstract

Halophyte *Lobularia maritima LmSAP* encodes an A20AN1 zinc-finger stress-associated protein which expression is up-regulated by abiotic stresses and heavy metals in transgenic tobacco. To deepen our understanding of LmSAP function, we isolated a 1,147 bp genomic fragment upstream of *LmSAP* coding sequence designated as *PrLmSAP*. *In silico* analyses of *PrLmSAP* revealed the presence of consensus CAAT and TATA boxes and *cis*-regulatory elements required for abiotic stress, phytohormones, pathogen, and wound responses, and also for tissue-specific expression. The *PrLmSAP* sequence was fused to the β-glucuronidase (*gusA*) reporter gene and transferred to rice. Histochemical GUS staining showed a pattern of tissue-specific expression in transgenic rice, with staining observed in roots, coleoptiles, leaves, stems and floral organs but not in seeds or in the root elongation zone. Wounding strongly stimulated GUS accumulation in leaves and stems. Interestingly, we observed a high stimulation of the promoter activity when rice seedlings were exposed to NaCl, PEG, ABA, MeJA, GA, cold, and heavy metals (Al^3+^, Cd^2+^, Cu^2+^ and Zn^2+^). These results suggest that the LmSAP promoter can be a convenient tool for stress-inducible gene expression and is a potential candidate for crop genetic engineering.

## Introduction

Agricultural production and food security are seriously impaired by various environmental stresses worldwide. To sustainably feed more than 10 billion people by 2050, food production requires rising by at least 70% [1]. Moreover, 20% of arable land is currently affected by environmental stresses, which affect crop yields and productivity and make the development of stress-tolerant crops more important [2].

Studies of promoters that largely regulate gene expression at the transcriptional level are crucial for improving our basic understanding of gene regulation and will expand the toolbox of available promoters for use in plant biotechnology. Promoters used in biotechnology applications can be grouped into the following classes: (1) Constitutive promoters (2) Spatio-temporal promoters and (3) Inducible promoters. The first class is active in all tissues at all times; the second one provides tissue-specific or stage-specific expression and the last type are promoters regulated by biotic and abiotic stresses, phytohormones, or external chemical stimuli. In crop genetic engineering, constitutive promoters or stress-inducible promoters are commonly used to test the function of genes in abiotic stress tolerance. Constitutively active promoters like cauliflower mosaic virus *CaMV 35S* [3], rice actin 1 (*Actin1*) [4], and the maize ubiquitin 1 (*Ubi-1*) promoters [5] have been widely used to control genes that confer both biotic and abiotic stress tolerance. However, strong constitutive expression of transgenes can be detrimental to the host plant, as several independent reports have reported severe reductions in seed production, growth retardation, altered grain composition and gene silencing, among other undesirable phenotypes of transgenic plants [6–8]. Tissue-specific or inducible promoters, by restricting gene expression to a particular tissue, developmental stage or to a specific stress response, may help to overcome these drawbacks.

Numerous stress-inducible promoters of many plant species have been characterized and functionally tested; some of these such as the stress-inducible *Arabidopsis* promoters, RD29A and COR15a, successfully minimize the adverse effects of constitutive transgene expression in tobacco, sugarcane, potato, *Chrysanthemum*, peanut, soybean and rice [9–15]. However, one of the most complex challenges for transgenic research today is the engineering of crops capable of coping with the simultaneous occurrence of multiple stresses (both biotic and abiotic). One strategy to address this challenge is to use inducible promoters with a combination of *cis* regulatory elements (motifs) that allow coordinated transcriptional control of multiple (trans) genes [16].

Salt-tolerant plants, or halophytes, have proven to be an interesting source of "tools" for the development of crops with better tolerance to abiotic stress and economically beneficial characteristics, being of particular interest for the isolation and characterization of abiotic stress tolerance genes and their respective inducible promoters. In the last two decades, various stress-responsive genes from halophytes have been characterized and their *cis*-regulatory motifs examined [17–19]. Thus, promoters of *T*. *halophila ThVP1* (*vacuolar H+-pyrophosphatase*) gene [20] and *AcBADH* (*Betaine Aldehyde Dehydrogenase*) from *Atriplex centralasiatica* [21] are highly induced by salt stress. The promoter of *CBL1* gene isolated from *Ammopiptanthus mongolicust* is inducible under both abiotic and biotic stress conditions [22]. The promoter of the *GSTU* (*Glutathione-S-Transferase*) gene from *Salicornia brachiata* contains abiotic stress-responsive *cis*-regulatory motifs [19].

In addition, among families of stress-responsive genes attracting interest, members of the Stress-Associated Proteins (SAPs) have been shown to impart tolerance to multiple abiotic and biotic stresses in transgenic plants [23–25]. The promoter of the *AlSAP* gene from the halophyte *Aeluropus littoralis* is inducible by abiotic stress, and expressed in an age-dependent, and tissue-specific pattern, thus representing a potential tool for engineering stress tolerance in crop species [17, 26]. Mishra and Tanna [27] argued that promoters from halophytes are promising candidates for genetic engineering due to the high stress-induction of driven genes. In previously published works, our group reported the characterization of the first *LmSAP* gene of the halotolerant plant *L*. *maritima*, which is induced by abiotic and heavy metal stresses, and whose overexpression in tobacco plants resulted in a higher tolerance to these stresses [24, 28]. In this study, the putative promoter region (1147-bp upstream from ATG) of *LmSAP* gene (named *PrLmSAP*) was isolated from *L*. *maritima* and tested in transgenic rice seedlings for analyzing its ability to control the expresstion of *gusA* gene under abiotic stresses and wounding. We showed that *PrLmASAP* is an active promoter, organ-specific and inducible by environmental stresses and wounding in transgenic rice. Overall, the inducible *PrLmSAP* promoter could potentially be used in crop biotechnology aiming at engineering tolerance to environmental stresses.

## Materials and methods

### Plant materials

Seeds of *L*. *maritima* were collected from salt marshes near Borj Cedria, a locality close to the Mediterranean seashore, 20 km North of Tunis. The rice cultivar *Oryza sativa* L. japonica cv. ‘Nipponbare’ was used for plant transformation (rice seeds were obtained from CIRAD-UMR AGAP, Plant Development and Genetic Improvement, Montepellier-France).

### Isolation of *PrLmSAP* by HE-TAIL-PCR method

The isolation of the 5’-flanking region of *LmSAP* gene was performed using HE-TAIL (High-efficiency thermal asymmetric interlaced) PCR method described by Michiels et al. [29]. Genomic DNA extracted from *L*. *maritima* leaves was used as template to carry out PCR reactions with four gene-specific reverse primers (LmSAP-Rev1: 5’-CGTTGAAACATTTCTGACACATGTTGTTCG-3’), (LmSAP-Rev2: 5’- CGCTGTAACGCCGCAGTTATTGGTACTT-3’), (LmSAP-Rev3: 5’- GAAGTTGAAGTCCTCTTGGAATCGAGTT-3’), and (LmSAP-Rev4: 5’- ACTCGCTCTCTACGTACTCCGAACTCTG-3’) designed close to the 5’ of *LmSAP* gene sequence, and four arbitrary degenerate primers (Rn1: 5’-NGTCGASWGANAWGAA-3’, Rn2: 5’- GTNCGASWCANAWGTT-3’, Rn3: 5’-WGTGNAGWANCANAGA-3’ and Rn4: 5’- NCAGCTWSCTNTSCTT-3’). Thermal conditions and reaction mixture of three rounds of PCR were used as described earlier by Michiels et al. [29]. Target amplification was purified and cloned in the pGEM-T Easy vector (Promega) and then recombined plasmids pGEM-*PrLmSAP* were transformed into *E*. *coli*. Positive clones were verified by PCR using universal primers. The promoter sequence of 1,147 bp, named *‘‘PrLmSAP”*, was obtained by sequencing and deposited in GenBank (NCBI ID: MK634698). Potential functional elements in the *PrLmSAP* sequence were identified using the software packages PLACE (https://sogo.dna.affrc.go.jp/cgi-bin/sogo.cgi?action=userInfo&lang=ja) and PlantCARE (http://bioinformatics.psb.ugent.be/webtools/plantcare/html; [30, 31]). Promoter sequences (the region around the 1.5 kbp upstream of the start codon) in *OsETHE1* (LOC_Os01g47690), *Nramp5* (LOC_Os07g15370), *Lsi1* (LOC_Os02g51110), and *WOX11* (LOC_Os07g48560) genes of *O*. *sativa* were retrieved from the RGAP database. The tool PLACE (https://www.dna.affrc.go.jp/PLACE/?action=newplace) was used for scanning of *cis-*regulatory elements present in promoter regions of *LmSAP* gene and four selected *O*.*sativa* genes.

### Cloning and rice transformation

*PrLmSAP* was released from pGEM-*PrLmSAP* via double digestion with *Spe*I/*Nco*I and subcloned in fusion with *gusA* gene in the binary vector pCAMBIA1301 vector (Cambia, Canberra, Australia), previously linearized with *Xba*I/*Nco*I restrictions enzymes to replace the CaMV-35S promoter. The construct pCAMBIA1301-*PrLmSAP*: *gusA* obtained and the pCAMBIA1301-CaMV35S: *gusA* were transformed into *Agrobacterium tumefaciens* EHA105 strain by the freeze-thaw method [32, 33] and used for transformation of rice due its easy and efficient method of transformation and regeneration. *Japonica* rice cv. Nipponbare seed-embryo-derived calli were transformed with the two constructs according to the previously described protocol [34]. Transgenic plants T0, named *PrLmSAP*: *gusA* and 35S: *gusA*, were selected on MS agar medium including hygromycin (50 mg/l). The transgenic plants T0 were transplanted into soil and allowed to self-fertilize to produce seeds of T1 and then T2 generation transgenic homozygous plants that were used in the different assays. The non-transgenic rice plants (NT) and 35S: *gusA* transgenic plants were used as negative and positive controls, respectively.

### Southern, northern and western blot analyses

Proper integration and expression of transgenes were verified by Southern and Northern blot hybridization in the T2 generation. For Southern blot, the genomic DNA was extracted from leaves of non-transformant (NT) and transgenic rice lines according to the protocol described by Gawel and Jarret [35]. A total amount of 5 μg genomic DNA was digested overnight with *BamH*I (cutting once in T-DNA) and separated through a 1% agarose gel. To perform Northern blot analysis, total RNA was extracted from 10 days of germinated seeds, roots, and leaves of *L*. *maritima*, transgenic rice lines, and NT seedlings using the Trizol reagent (Invitrogen, Carlsbad, CA, USA) according to the manufacturer’s recommendations. A total amount of 10 μg of total RNA samples was separated on a 1.5% formaldehyde gel. DNA and RNA membranes were transferred onto a Hybond-N^+^ membrane (Amersham Biosciences). The *LmSAP* cDNA fragment and the *gusA* cDNA fragment amplified by PCR with a specific primers (qLmSAP-F: 5’-AAGGGATGTTCTGGTTGTCG-3’; qLmSAP-R: 5’-TGATGACGATCGGAGTAACG-3’; GUSF: 5’-CTCCTACCGTACCTCGCATTAC-3’; and GUSR: 5’- ACGCGCTATCAGCTCTTTAATC-3’) and then labeled with [α-^32^P] dCTP (Amersham Biosciences) were used as a probe. The hybridization was accorded by the protocol described by Ben Saad et al. [17].

For immunoblot analyses, total proteins were extracted from 0.1 g of four transgenic rice lines (*PrLmSAP*–*gusA*: L6, L20, L13, and L10) and NT plants in 3 ml of extraction buffer [50 mM Tris–HCl (pH 8), 10 mM MgCl_2_, 1 mM EDTA 0.5 M (pH 8), 5% glycerol, 1 mM DTT, 0.1% Triton X-100 and 1 mM (PMSF)]. The homogenate was placed on ice for 5 min, and then centrifuged at 13,000 *× g* for 15 min. The proteins concentrations of the resulting supernatant were determined by the Bradford method [36]. Total proteins samples were then electrophoretically separated on 12% (w/v) SDS–PAGE gels and blotted by the semi-dry method (iBlotTM Gel Transfer Stack) onto a nitrocellulose membrane (Invitrogen). GUSA protein detection was carried by the same protocol described by Ben Saad et al. [17].

### Stress treatments of transgenic rice plants

To elucidate the *PrLmSAP* promoter activity during the earliest stages of plant development, seedlings of two homozygous transgenic lines (L6-*PrLmSAP*::*gusA* and L13-*PrLmSAP*:: *gusA*) were stained for 12, 48 and 72h after germination. To evaluate the effect of various stress treatments, transgenic seedlings harboring the LmSAP promoter (L6-*PrLmSAP*::*gusA* and L13-*PrLmSAP*::*gusA*) were submerged in aqueous 1/2 MS medium supplemented with 150 mM NaCl, 10% PEG-6000, 50 μM gibberellin (GA), 100 μM abscisic acid (ABA), 100 μM Methyl jasmonate acid (MeJA), 100 μM CuSO_4_, 100 μM ZnSO_4_, 50 μM CdCl_2_ or 50 μM AlCl_3_ for 24h. For cold treatment, seedlings were stressed at 4°C for 24h. Seedlings immersed in 1/2 MS medium for 24h without any other treatment were appointed as control. All experiments were conducted with three technical replications 7 days after seed germination (DAG). To determine *PrLmSAP* promoter activity during wounding, old leaves of transgenic rice plants were injured with a blade and stained 2 h after wounding with X-gluc solution for GUS assays.

### Histochemical localization and quantitative analysis of GUS activity

The GUS staining was processed as mentioned above by Ben Saad et al. [26]. Before photographing or performing histological analyzes the stained samples were soaked in 70% ethanol to remove chlorophyll. GUS staining was observed with a Leica MZFLIII binocular microscope (Leica Microsystems, Heerbrugg, Switzerland). Transversal sections of 20 μm thickness were cut using a vibratome after mounting root and leaf tissues in 4% (w/v) agarose blocks. The observations of histological tissue sections were accomplished by using a microscope BH2 (Olympus) under white light. GUS activity was analyzed quantitatively in 10 day-old transgenic L6-*PrLmSAP*::*gusA* and L13-*PrLmSAP*::*gusA* seedlings using 4-methyl umbelliferyl glucuronide (4-MUG) according to the method mentioned by Jefferson et al. [37]. The GUS specific activity was measured at 460 nm via Fluoroscan (Labsystems) apparatus operating. The GUS specific activity is expressed as microgram’s of 4-methylumbelliferone (4-MU) per minute per milligram protein. In order to ensure reproducibility, each assay was performed three times.

### Statistical analyses

The data for all experiments are presented as the mean ± standard error (s.e) of three replicates. Statistical analyses were performed using the XLSTAT software with one-way ANOVA. Mean values marked with different lowercase letters on the figures are significantly different at *P* < 0.05 according to Bonferroni’s *post hoc* test.

## Results

### Isolation and bioinformatic analysis of *PrLmSAP* promoter

**Fig 1:**
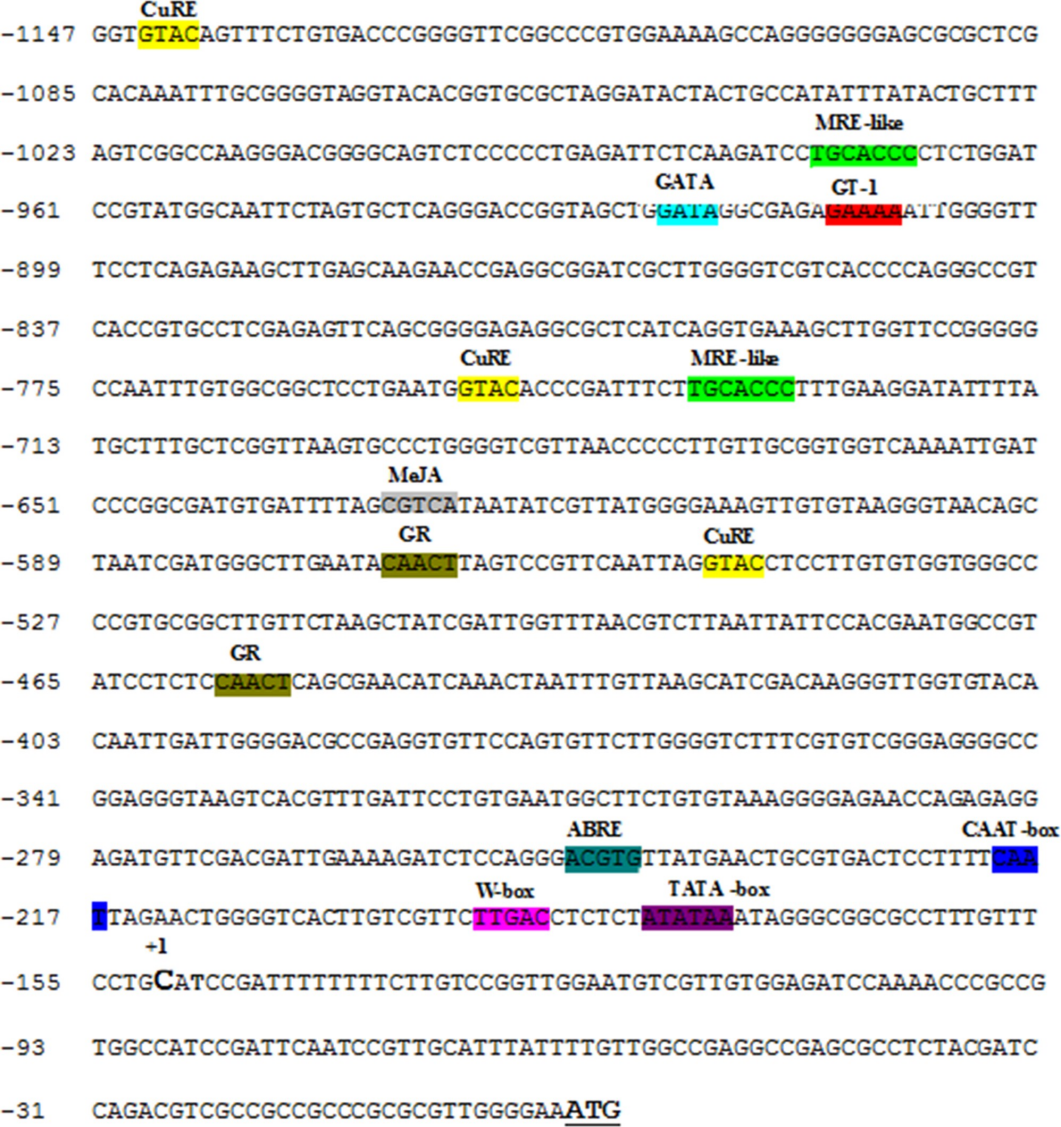
Pictorial representation of cis-regulatory motifs on LmSAP promoter. The putative transcription start site is denoted +1; the start code (ATG), position of cis-motifs on promoter sequence are represented with colourful highlight and positioned upstream to the translation start site. CuRE: copper response element; MRE: metal-response elements (MREs: 5’-TGCRCNC-3’ (R = A or G; N = any residue); GR: Gibberellin responsive; MeJA: Methyl jasmonate acid responsiveness; ABRE: ABA responsiveness. Remaining abbreviation used here is the canonical *cis*-element names.

The *PrLmSAP*—a 1,147 bp fragment upstream the *LmSAP* coding sequence—was isolated from *L*. *maritima* genomic DNA by the TAIL-PCR technique [29]. During the tertiary PCR reaction, two specific bands of 1,180 and 1,147 bp length were amplified using the LmSAPRev3/Rn2 and LmSAPRev4/Rn2 primers, respectively, cloned and then sequenced. This putative *LmSAP* promoter sequence was subjected to homology-based *in-silico* analyses using PLACE and PlantCARE programs, in order to predict the *cis*-acting elements (Fig 1).

Analysis of this sequence using Proscan software (http://www-bimas.cit.nih.gov/cgi-bin/molbio/proscanUH) showed the presence of putative transcription start-site (TSS) at base 150 upstream the ATG start codon, and the position is marked as +1. A hypothetical TATA-box was identified at -30 bp relative to the TSS (Fig 1) and a CAAT-box at -69 bp, which were consistent with the regular features of eukaryotic promoters [38] and primordial for the transcription initiation and basal levels of transcription (Fig 1, Table 1). Several *cis*-elements were identified in the *PrLmSAP* sequence that included elements related to the response to hormones such as abscisic acid (ABA), salicylic acid, gibberellin, auxin and methyl-jasmonate (MeJA), and responsiveness to physiological and environmental stresses, such as salt, drought, light, dehydration, wounding, and pathogen/elicitor. *Cis*-elements important for temporal and spatial gene expression during different developmental stages, such as seed, pollen, mesophyll and nodules were also found (Table 1). Interestingly, two MRE-like sequences were located at positions (−1077/−1070 and−821/−814) in the *PrLmSAP* sequence, close to animals core MRE motifs reported to be implicated in the heavy metal-induced expression of metallothionein genes (*MTs*) [39]. Three CuRE elements, another heavy metal *cis*-element, were also identified at the positions (−1144/−1141, −784/−780 and −632/−628) (Fig 1) [40, 41]. The eventual presence of these stress-related *cis*-elements suggests that *PrLmSAP* might react to phytohormones, wounds, heavy metals, and numerous environmental stresses (Table 1).

**Table 1:** Different *cis*-regulatory motifs of *LmSAP* gene promoter predicted by an online program.

Category	*Cis*-element name	Function	Position
**Pathogen, elicitor and wound responsive**	**WRKY710S**	W box of transcriptional repressor gene of gibberellin signaling pathway, also MYB binding	-485 (-), 517(-) - 917 (+), -942 (-) - 957(+), - 146 (-) - 304 (-)
	**WBOXNTCHN48**	W-box found in the chitinase 1 and 2 gene for elicitor responsive expression	-504(+)
	**W-box**	Wounding-responsive	-483(-), -955(+)
	**WBOXNTERF3**	W box, wound signal responsive	-516(-), -917(+), -941(-), -957(+)
	**BIHD10S**	BELL like homeodomain transcription factor binding site in response to pathogen attack	-155(-)
	**ELRECOREPCRP1**	EIRE (Elicitor response element)	-484(-)
**ABA, Dehydration and salinity stress responsive**	**ABRERATCAL**	ABRE-related sequence" or "Repeated sequence motifs" identified in the upstream regions of 162 Ca2+ responsive up regulated genes	-669(-), -1135(-), -1049(-), -1307(-)
	**MYB1AT**	MYB recognition site found in the promoter of dehydration responsive gene rd22	-457(-)
	**MYC CONSENSUSAT**	MYC recognition site found in promoter of dehydration responsive genes	-349(-), -349(+), -944(-), -1283(+) -1248(+), -1248(-)
	**ANAERO2CONSENSUS**	One of the 16 anaerobic stress responsive motifs	-986(-), -1196(+)
	**CBFHV**	Cold responsive DRE (binding site of HvCBF1)	-126(-), -724(+), -795(+)
	**WBOXATNRP1**	Salicylic acid responsive	-956(+)
	**GT1GMSCAM4**	GT-1 Salt and pathogen inducible	-444(+)
	**ACGTATERD1**	Etiolation induced expression of erd1	-671(-), -671(+), -900(-), -900(+), -1282(-), -757(+), -1065(+), -757(-), -1065(-)
	**CURECORECR**	Oxygen deficiency responsive gene expression through copper-sensing signal transduction pathway	-4(-), -4(+), -397(-), -397(+), -599(+), -599(-)
	**DPBFCOREDCDC3**	ABA inducible bZIP transcription factor DPBF-1 and 2 binding site	-606(-)
	**DRECRTCOREAT**	Dehydration-responsive element/C-repeat	-126(-)
	**TC-rich repeats**	*Cis-acting* element involved in defense and stress responsiveness	-749(-)
**Phytohormone responsive**	**ARR1AT**	Cytokinin regulated ARR1 binding site	-1063(+), -1069(-), -1001(+)
	**CAREOSREP1**	Gibberellin regulated proteinase expression	-691(+)
	**ABRE**	ABA responsiveness	-818(-), -900(+)
	**TGA-element**	Auxin-responsive element	-25(+), -1027(-), -948(-)
	**GCCCORE**	Ethylene responsive element	-1125(+), -1128(+)
	**CGTCA-motif**	MeJA-responsiveness	-307(+)
**Tissue/cell specific**	**OSE2ROOTNODULE**	Nodule and organ specific expression after infection	-886 (+)
	**NODCON1GM**	Nodule specific expression	-886 (+)
	**ROOTMOTIFTAPOX1**	Root specific motif	-522(-), -663(-), -664(+), -811(+)
	**RAV1BAT**	Leaf and root specific	-349 (-)
	**CAATBOX1**	Seed specific	-404 (+), -491(-), -640(+), -928(+), -1068(+)
	**POLLEN1LELAT52**	Pollen specific activation	-8 (+), -407 (-), -470 (-), -744 (+), -749 (+), -1009(-)
	**GTGANTG10**	GTGA motif in late pollen gene g10 promoter	-34 (+), -147(+), -310 (-), -352 (+) -390 (+), -506 (+)
	**GATABOX**	Leaf and shoot specific	-190(-), -524(-), -682(-), -813(-)
	**TAAGSTKST1**	Dof protein regulating guard cell specific gene expression	-594(+)
	**CACTFTPPCA1**	Mesophyll specific expression in C4 plants	-1(-), -33(-), -99(-), -103(-), -116(-), -158(-), -202(-)
	**DOFCOREZM**	Core site required for binding of Dof proteins in maize	-123(-), -158(+), -155(-), -195(-), -213(+), -240(+), -286(-), -367(-), -418(-), -418(-), -437(-)
**Light responsive**	**ASF1MOTIFCAMV**	Light induced auxin and salicylic acid regulated as-1 motif	-308(-)
	**EBOXBNNAPA**	E-Box drive light responsive expression	-349(+), -879(-), -944(-), -944(+)
	**GATABOX**	Light responsive (found in promoter of all LHCII type I Cab genes	-224(+)
	**GT1CONSENSUS**	GT-1 motif; Light regulated expression	-1007(-), -235(+), -240(+), - 425(+)
	**G-BOX**	Light responsive elements	-898(-)
	**GT1CORE**	Light responsive elements	-445(+)
**Conserved promoter motifs**	**CAATBOX1**	CAAT promoter consensus sequence	-650(+), -717(-), -878(+),-712(-), -648(+)
	**TATABOX4**	TATA box found in the 5 'upstream region of sweet potato sporamin A gene	-966 (+)
	**TATABOX5**	TATA Box found in the 5’ upstream region of pea (*Pisum sativum*) glutamine synthetase gene	-1081 (+)

### Generation of *PrLmSAP*:: gusA transgenic rice lines

**Fig 2:**
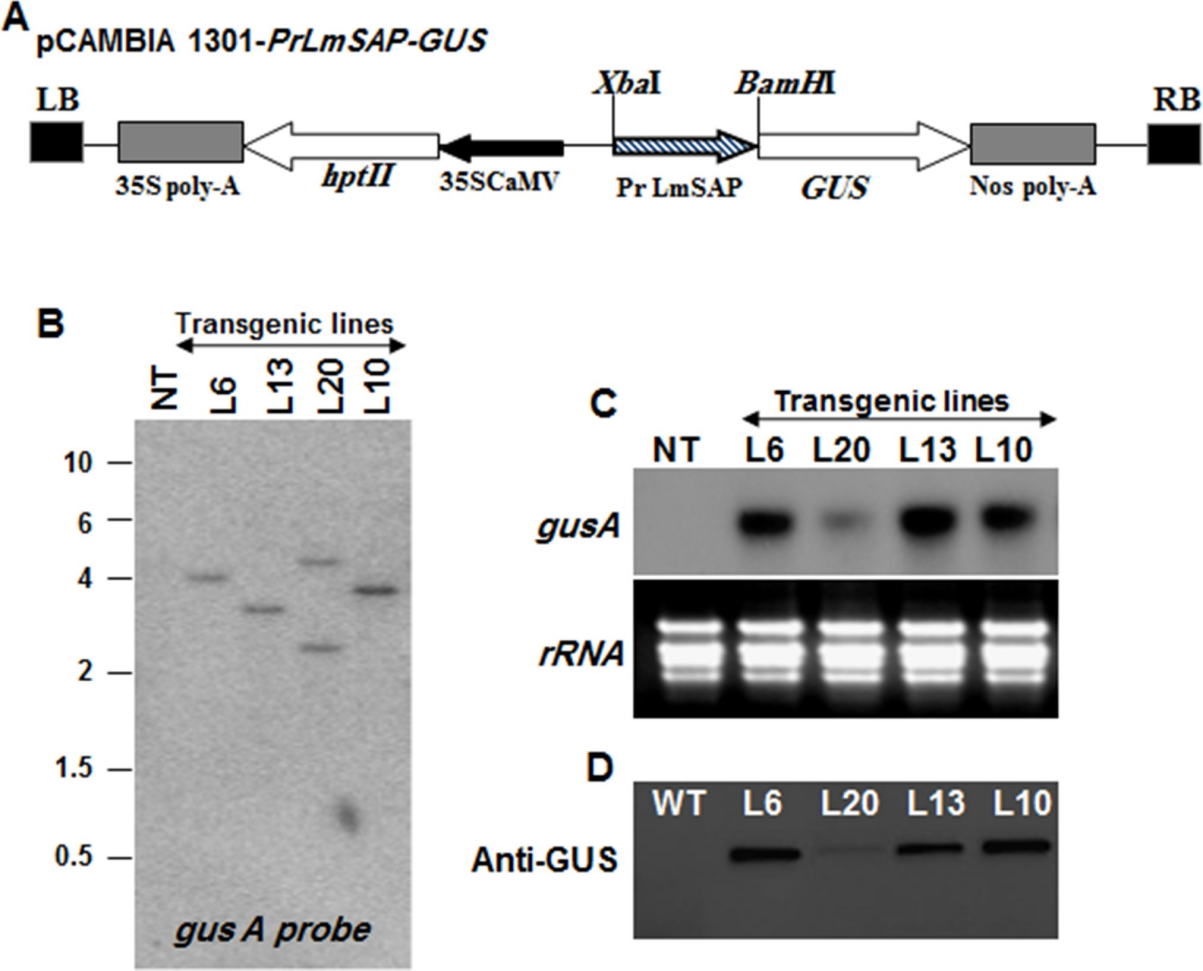
Analysis of transgenic rice lines expressing the *gusA* gene under the *PrLmSAP* promoter. **(A)** Schematic representation of the T-DNA cloned in the pCambia1301 binary vector and used for rice transformation. *PrLmSAP*::*gusA* the stress-inducible promoter *PrLmSAP* fused to *gusA* gene. **(B)** Determination of the T-DNA copy number in transgenic and NT rice plants via southern blot analysis. **(C)** Northern blot analysis of *gusA* transcript levels in NT and the transgenic lines of rice. **(D)** Western blot analysis using the anti-GUS antibody to detect the presence of GUS protein in transgenic rice lines.

The *GUS* reporter gene was combined to the promoter sequence and then used to transform rice (Fig 2A). Several independent transgenic rice lines were obtained and the presence of the *PrLmSAP*::*gusA* fusion was verified by PCR from genomic DNA. Both the number of copies integrated into the transgenic lines and the *gusA* gene transcription levels were confirmed in four transgenic lines (L6, L13, L20 and L10) by Southern and Northern blot hybridization, respectively (Fig 2B and 2C). These lines stably express the *gusA* transgene at variable levels (Fig 2D). Lines L6, L13 and L10 possess one T-DNA insertion and exhibit high GUS protein level whereas line L20 has two T-DNA insertions and accumulates less GUS protein. L6, L13 and L10 were grown and followed until T2 homozygous seeds were obtained. Two representative transgenic lines (L6 and L3) were used to explore the *PrLmSAP* promoter's functional properties.

### *PrLmSAP* is a tissue-specific promoter in transgenic rice

**Fig 3:**
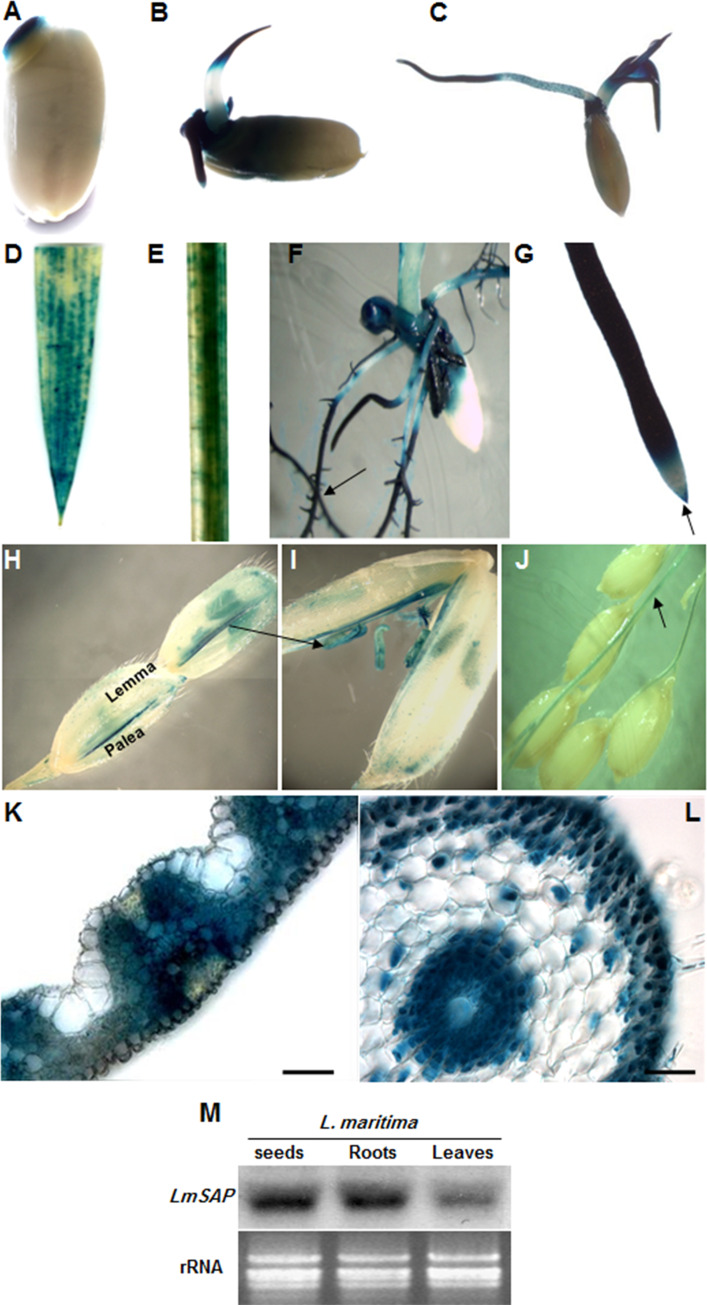
The histochemical localization of *β*-glucuronidase (GUS) activity in representative transgenic rice plants harboring L6-*PrLmSAP*::*gusA* during seed germination, in flowers and panicle. (**A–C**) Seedlings grown on MS medium at 12h (**A**), 24h (**B**) and 72h (**C**). **(D)** Leaf, **(E)** Stem, **(F)** Lateral root, **(G)** Seminal root, (**H–J**) Reproductive organs of the transgenic plants (**H-I**, mature flower; **J**, panicle). (**K)** Transversal vibratome section through a leaf blade of transgenic rice harboring the *PrLmSAP*::*gusA*. (**L)** Transversal vibratome section through the seminal root of transgenic rice harboring the L6-*PrLmSAP*::*gusA*. Blue precipitates indicate positive GUS signals. Bars 50 μm. (**M**) Northern blot analysis of *LmSAP* gene in pivotals organs (germinated seeds, leaves, and roots) of *L*. *maritima*. RNA was transferred onto a nylon membrane and hybridized with the [α-32P] dCTP labeled *LmSAP* full-length cDNA probe. Equal loading in each lane was confirmed by ethidium bromide staining (lower panel).

Histochemical GUS staining of transgenic rice line (L6) expressing the *gusA* reporter gene under the control of the *PrLmSAP* was carried out throughout plant development. As shown in Fig 3, *PrLmSAP* was active very early after seed germination and throughout the seedling stage. Indeed, intense GUS staining was detected 12, 48 and 72 h after germination in the germinating embryo axis, coleoptile and seminal root elongation zone (Fig 3A and 3C). The GUS staining was stronger in roots (Fig 3F and 3G) but relatively weak in leaves and stems (Fig 3D and 3E). Roots of L6-*PrLmSAP*::*gusA* seedlings exhibited strong GUS staining in the root apical meristem and in distal parts of the primary root as well as at the branching sites of lateral roots but it was absent from the division zone (Fig 3F and 3G). We also stained whole flowers to study GUS expression in the reproductive organs of the transgenic rice line, detecting high GUS activity in stamens, pistils and rachis, but not in the lemma and palea (Fig 3H and 3J); immature seeds were not stained indicating that *PrLmSAP* is not active in these tissues or developmental stage (Fig 3J). To precisely define the tissue-specific location of *PrLmSAP* activity in L6 rice line, histological leaves and roots sections were prepared using a vibratome. Transversal cross-sections of leaf blades from *PrLmSAP*::*gusA* seedlings showed a strong GUS activity in the mesophyll, phloem and xylem cells, while little staining was observed in the upper and lower epidermis (Fig 3K). Transversal sections of the seminal root, where cellular maturation occurs, showed strong GUS staining in the epidermis, exodermis, endodermis, metaxylem and phloem, while slight GUS staining was observed in sclerenchyma cortex (Fig 3L). It is important to note that same results for GUS expression were showed in transgenic seedlings of the line L13 harbouring the *PrLmSAP*::*gusA* construct (S2A–S2L Fig). In order to investigate the tissue-specific expression patterns of *LmSAP* transcript in the various tissues of *L*. *maritima* seedlings, total RNA from the frozen samples was isolated from 10 days of germinated seeds, leaves, and roots. Northern blot analysis showed that the same level of *LmSAP* transcript in germinated seeds, roots, and leaves (Fig 3M) which correlated with the tissue specificity of the *LmSAP* promoter. These results show that the *PrLmSAP* promoter is important for precise developmental regulation in both the vegetative and reproductive organs.

### Environmental stresses and wounding induces *PrLmSAP* promoter

**Fig 4:**
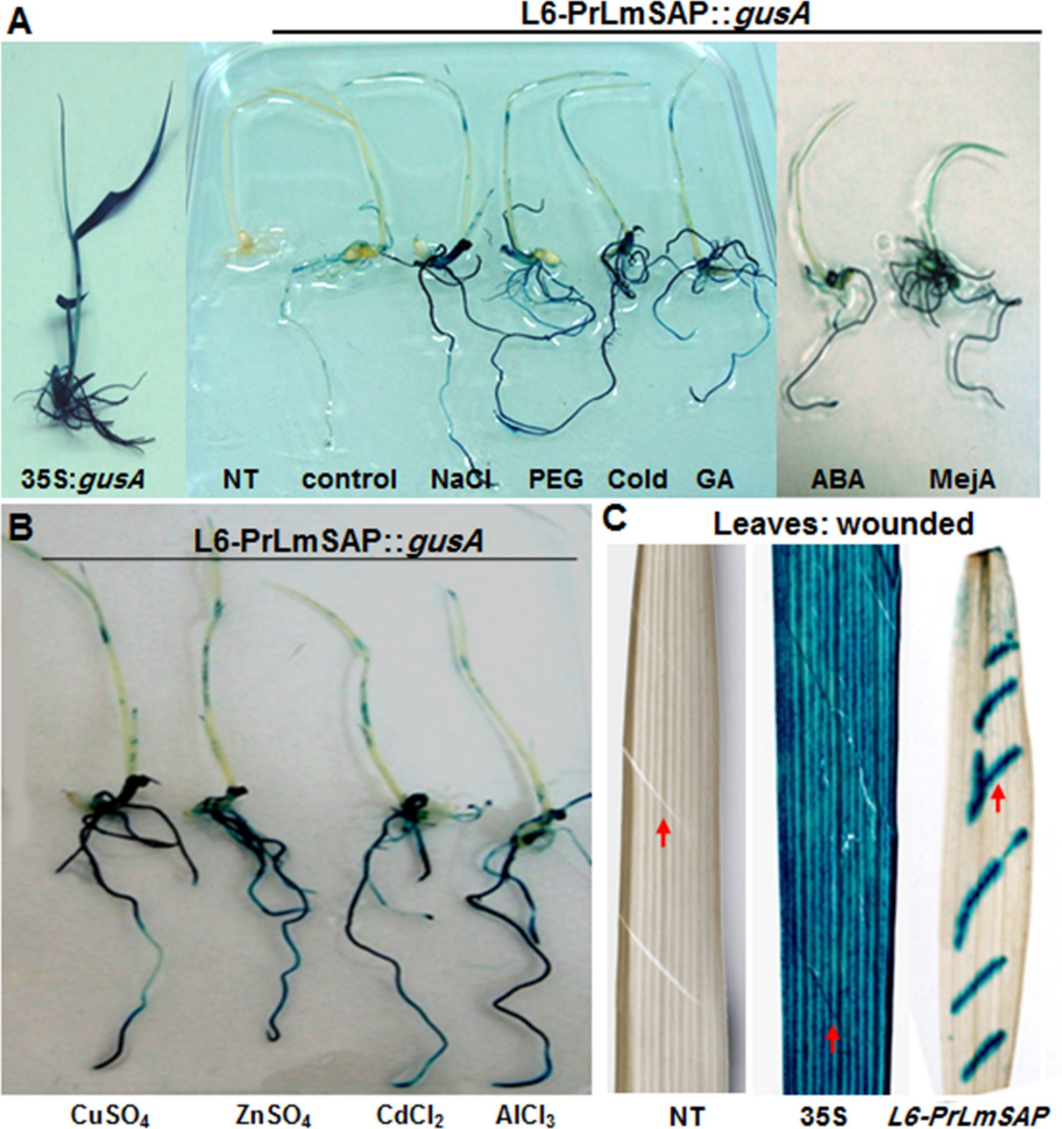
Induction by abiotic stress treatments of the *PrLmSAP* promoter in transgenic rice. **(A)** Histochemical assays on representative 7 DAG (days after germination) seedlings stressed with NaCl (150 mM), 10% PEG-6000, cold (4°C), GA (50 μM), ABA (100 μM) or MeJA (100 μM) for 24h. **(B)** Histochemical assays on representative 7 DAG (days after germination) seedlings stressed with CuSO_4_ (100 μM), ZnSO_4_ (100 μM), CdCl_2_ (50 μM) or AlCl_3_ (50 μM) for 24h. **(C)** Detection of GUS in L6-*PrLmSAP*::*gusA* transgenic rice leaves following wounding. NT plants (negative control), transgenic rice plants expressing the *GUS gene* from the constitutive 35S promoter (positive control) and L6-*PrLmSAP*::*gusA* Transgenic plants were developed by transforming the promoter of *LmSAP* gene (red arrows highlight wound sites).

Recently, we showed that the expression of the *LmSAP* gene in *L*. *maritima* is induced by abiotic stresses and heavy metals [24, 28]. In the present analysis of the *PrLmSAP* promoter, we revealed the presence of different motifs related to environmental stresses (Table 1). The quantitative GUS activity assay showed that tissues of stressed rice seedlings exhibited stronger GUS accumulation than those grown under control conditions (Fig 4A); especially in the roots (Fig 4A and 4B). In contrast, GUS staining was not detected in NT plants while the CaMV35S::*gusA* transgenic lines exhibited a dark blue staining overall the seedlings (Fig 4A). Following heavy metal exposure, a strong GUS activity was detected in the roots of transgenic rice seedlings harbouring the *PrLmSAP*::*gusA*, compared to seedlings grown under control conditions (Fig 4B). Similar GUS expression patterns were observed across the L13-*PrLmSAP*::*gusA* transgenic line stressed with different abiotic stresses (S3 Fig). These results show that the *PrLmSAP* promoter is induced by diverse environmental stresses, especially in roots. Furthermore, we also tested wounding by mechanically injuring the leaves of L6, CaMV35S::*gusA* (positive control) and NT (negative control) lines. Strong GUS staining was detected at wounded sites in L6, whereas leaf tissues from CaMV35S::*gusA* plants exhibited deep blue staining even in the absence of wounding (Fig 4C). As expected, no GUS activity was detected in NT plants (Fig 4C). These results clearly demonstrate that wounding can activate *PrLmSAP*.

### Fluorometric analysis of GUS activities in transgenic rice under various environmental stresses

**Fig 5:**
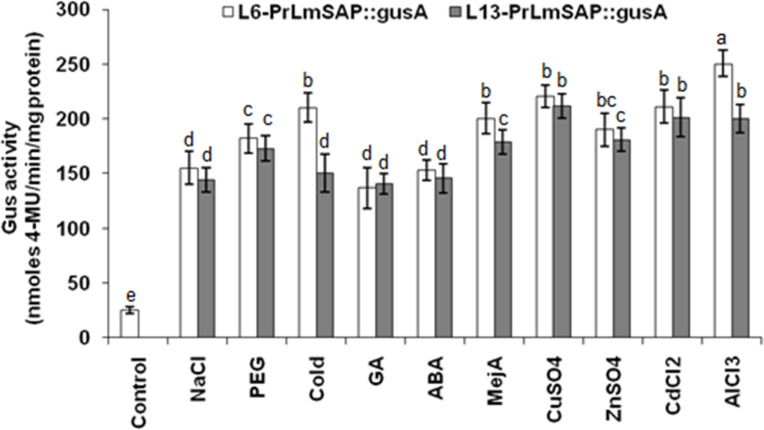
Changes of *GUS* expression in response to various stresses in L6-*PrLmSAP*:: *gusA* and L13-*PrLmSAP*::*gusA* transgenic rice plants. For each treatment, GUS fluorescence was measured in 10 day-old seedlings. GUS activity is expressed as nanomoles of 4-methylumbelliferone (4-MU) per minute per milligram protein. Numbers above the bars showed the change fold of various external stresses over untreated control seedlings. The data represent the mean ± SE of three replicates. Means denoted by the same letter were not significantly different at *p* < 0.05.

Our work clearly shows that different types of stress activate *PrLmSAP*. Thus we measured the GUS activity on 10-day old T2 seedlings of two independent *PrLmSAP*::*gusA* lines (L6 and L13) treated with various stress treatments, in order to have a more quantitative measure of its activation. As shown in Fig 5, GUS activity in L6-*PrLmSAP*::*gusA* increased markedly when seedlings were stressed by cold (8.41 fold), PEG (7.28 fold), or NaCl (6.28 fold) as compared with untreated plants (control). A strong induction was also observed after treatment with MeJA (8.02 fold), ABA (6.13 fold) and GA (5.46 fold). Concerning heavy metals, Al^3+^ induced the highest GUS activity, with a 10-fold increase compared to control seedlings, followed by Cu^2+^ (8.81 fold), Cd^2+^ (8.45 fold) and Zn^2+^ (7.6 fold) (Fig 5). These results indicate that this diverse range of environmental stresses may regulate *LmSAP* expression through the identified *cis*-acting elements found in its promoter. The activation of *PrLmSAP* by these numerous and varied type of stresses places it as a good candidate for regulating the expression of genes involved in enhancing plant responses to abiotic and biotic stresses.

### *In silico* comparative analysis of *PrLmSAP* with other *SAP*- and root-specific gene promoters

In order to have more information about the number of predicted regulatory elements in *PrLmSAP*, we compared approximately 1000-bp genomic sequences upstream of the start codon from other *SAP* gene promoters of *Arabidopsis thaliana* (*PrAtSAP5* (*At3g12630*), *PrAtSAP13* (*At3G57480*)) and *Oryza sativa* (*PrOsSAP9* (*Os07g07350*.*1*) (Table 2). *In silico* analysis of promoter sequences revealed that *PrLmSAP* has the highest number of wounding responsive elements, etiolation induced expression, early responsive elements in dehydration, auxin-responsive elements, tissue-specific expression elements, light-responsive expression and CAAT-box (Table 2).

**Table 2:** Number of putative *cis*-acting elements present in the promoter region of *LmSAP* were compared with others *SAP* genes of *Arabidopsis thaliana* (*PrAtSAP5* (*At3g12630*), *PrAtSAP13* (*At3G57480*)) and *Orysa sativa* (*PrOsSAP9* (*Os07g07350*.*1*) according to the PLANT CARE and PLACE databases.

Function	Motifs	PrLmSAP	PrAtSAP5	PrAtSAP13	PrOsSAP9
**Elicitor-responsive elemnt**	WBOXNTCHN48	1	1	2	0
	ELRECOREPCRP1	1	0	0	0
**wounding activation gene elements**	W-box	2	1	0	0
	WBOXNTERF3	4	1	1	0
***Cis-acting* element involved in defense and stress responsiveness**	TC-rich repeats	1	1	1	1
**Early responsive elements in dehydration**	ABRERATCAL	4	1	3	1
	ACGTATERD1	6	2	0	4
	DRECRTCOREAT	1	1	0	1
**GT-1 Salt and pathogen inducible**	GT1GMSCAM4	1	2	1	0
**ABA**	ABRE	2	1	1	1
**MYB (MYB binding site involved in drought inducibility)**	MYB1AT	1	2	1	1
**MYC (MYC binding site involved in dehydration responsive gene)**	MYC CONSENSUSAT	6	4	4	0
**Cold responsive DRE**	CBFHV	3	1	1	2
**Etiolation induced expression**	ACGTATERD1	9	0	2	0
**Salicylic acid responsive**	WBOXATNRP1	1	2	1	0
**Gibberellin-responsive element**	CAREOSREP1	1	1	1	2
**Auxin-responsive element**	TGA-element	3	0	0	0
**Ethylene responsive element**	GCCCORE	2	1	0	0
**MeJA-responsiveness**	CGTCA-motif	1	1	0	0
**Nodule specific expression**	OSE2ROOTNODULE	1	1	0	0
	NODCON1GM	1	1	1	1
**Root specific motif**	ROOTMOTIFTAPOX1	4	4	4	2
**Leaf and root specific**	RAV1BAT	1	2	1	0
	GATABOX	4	2	3	0
**Seed specific**	CAATBOX1	5	5	1	0
**Pollen specific activation**	POLLEN1LELAT52	6	0	1	0
**Tissue specific expression elements**	CACTFTPPCA1	7	5	5	2
**light responsive expression**	ASF1MOTIFCAMV	1	1	1	0
	EBOXBNNAPA	4	2	2	0
	G-BOX	1	0	0	1
	GT1CONSENSUS	4	4	4	0
**WRKY**	WRKY710S	7	6	5	0
**Promoter and enhancer**	CAATBOX1	5	4	3	1
**Core promoter element around -30 of transcription start**	TATABOX	2	2	2	1

We also compared the distribution of *cis*-regulatory elements present in *PrLmSAP* and four root-specific promoters highly expressed in rice (*PrOsETHE1*, *PrNramp5*, *PrLsi1*, and *PrWOX11*) [42]. The results indicated widely overlapped *cis*-regulatory elements between all promoter sequences analyzed (S1 Fig; S1 Table). The *cis*-regulatory elements recognized were involved in tissue-specific expression (Tef-box and root hair-specific cis-element), hormones responsiveness (GA-responsive element, ABA-responsive element, salicylic acid regulatory element, ethylene-responsive element), and transcriptional gene regulation (MYB recognition site). However, compared with the other root-specific promoters, *PrLmSAP* showed a more ubiquitous expression pattern in the roots (S1 Table) or/and stronger expression [42].

## Discussion

Stress-associated proteins (SAP) have been identified in the model species *Arabidopsis thaliana* and *Oryza sativa* [25, 43] but also in several non-model plants from harsh environments such as the halophyte species *Aeluropus littoralis* [23] and *Lobularia maritima* [24]. Through transgenic and cisgenic approaches, it has been demonstrated that overexpression of SAP-coding genes results in enhanced tolerance to drought, extreme temperatures, salinity, oxidative stress and metal toxicity [25]. Most of the *SAP* genes are tissue-specifically expressed and induced by stress conditions [23, 24, 44, 45], while some others are constitutively expressed [46]. These characteristics of *SAP* genes (responsiveness to multiple stresses, inducibility and tissue-specificity) made them interesting candidates for improving crop response to abiotic stress but also for identifying new promoter regions to assist transgenic approaches.

*Lobularia maritima* (L.) Desv., is a diploid (2n = 24) herbaceous perennial and facultative halophyte belonging to the clade C of the family Brassicaceae, closely related to *A*. *thaliana* [47]. Transcripts from *Lobularia* share in average 90% identity with homologous genes from *Arabidopsis* [48, 49]. In previous works, we showed that *LmSAP*, a gene encoding an A20/AN1 zinc-finger protein from *L*. *maritima*, plays a key role in tolerance to salt, ionic and metal stress in *L*. *maritima* and transgenic tobacco lines, potentially through ROS-scavenging and metal-chelation [28]. In this investigation, we presented the isolation of the *LmSAP* promoter region, named *PrLmSAP*, and its characterization by expression in transgenic rice, thus providing novel insights into understanding the transcriptional regulation of *SAP* genes and a potential new resource for crop genetic improvement by transgenic technology.

Analysis of the promoter sequences performed in this work revealed the presence of *cis*-regulatory elements responsive to multiple abiotic stresses, but also transcription factor-binding sites such as MYB, MYC, Dof, and WRKY and hormones responsive motifs. Some motifs responsible for tissue-specific expression and light-induced expression were also found (Table 1). The interaction between these *cis*-elements and specific transcription factors could lead to the activation of stress-associated genes involved in protecting plants against abiotic stresses.

Important elements found in *PrLmSAP* include the dehydration-responsive element DRE (A/GCCGAC) involved in the regulation of cold and dehydration responses in *Arabidopsis* [50], the low temperature-responsive element C-repeat binding factor (CBF) [51], and the ABA-responsive element ABRE (ACGTGG/T) that regulates dehydration and salinity responses in *Arabidopsis* and rice [52]. Analysis of the dehydration-responsive gene *RD29B* promoter, containing ABRE and DRE/CRT elements, proved that different abiotic stresses are extensively connected [53]. Moreover, our sequence analysis revealed that *PrLmSAP* contains one putative CARE (CAACTC) necessary and sufficient for GA-inducible expression. Many GA responsive sequences have been identified to date, including GAREs [54], GA response complexes (GARCs) [55], and CAACTC regulatory elements (CAREs) [56]. CAREs are involved in GA responsiveness and transactivation by GAMyb. Mutations of CAREs cause GA inducibility loss and block GAMyb transactivation, indicating that CAREs are regulatory elements involved in GA-inducible expression [56]. Besides, *cis*-elements involved in signalling- and responsiveness to the phytohormones auxin, ethylene, MeJA, and SA were also identified in *PrLmSAP*, supporting a putative involvement of LmSAP in plant defence against pathogens [25].

To understand the regulatory mechanisms controlling *LmSAP* gene expression, the expression pattern of *PrLmSAP* driving *gusA* reporter gene in transgenic rice plants was investigated. Both histochemical staining and histological sections revealed that the *PrLmSAP* promoter is induced by multiple abiotic stresses but in a tissue- or organ-specific manner. Moreover, GUS activity was detected at low levels in roots where the activity increased substantially after NaCl, PEG, cold, GA, ABA, MeJA, wounding, or heavy metals treatments.

Transverse sections of leaves and roots of *PrLmSAP*::*gusA* lines after histochemical staining showed higher-intensity GUS in conducting tissues of leaves and roots. Similarly, strong expression in vascular bundles was detected for other salinity responsive genes [57, 58]. In reproductive organs, GUS activity was observed in stigma, filaments, and anthers of mature flowers but not in the lemmas and palea of the *PrLmSAP*::*gusA*. Many anther- and pollen-specific promoters have been isolated from different plants, such as the *RA8* promoter from rice, the *A9* promoter from *Arabidopsis*, and the *TA29* promoter from tobacco which is an important trait for plant breeding [59]. These promoters have been successfully employed to regulate the expression of the barnase protein [60] and to control a cytotoxic protein in transgenic plants [61]. Moreover, *PrLmSAP* expression is active in the vascular tissues of both vegetative and reproductive organs. Since the vascular system is involved in water, nutrients and metabolite transport, this observation suggests that LmSAP may play a role in vascular transport.

We clearly showed that *PrLmSAP* is inducible by wounding. This result is consistent with the presence of wounding-related elements (W-box) may be an active player in controlling the rapid expression of *LmSAP* upon mechanical damage and involved in wound-induced signalling and recognized by the WRKY transcription factors [62, 63]. Recently, we reported that the expression of *LmSAP* gene was up-regulated after 12h of treatments with the heavy metals cadmium, copper, manganese and zinc, suggesting its involvement in plant responses to heavy metal stress [28]. Interestingly, two copies of MRE-like motifs present in animals and three copies of putative CuRE sequences have been identified in *PrLmSAP*. In agreement with this, Quinn et al. [64] reported that a copper response regulator, CRR1, binds to CuRE sites and mediates the expression of downstream genes under copper-deficient conditions. Another report proved that both CRR1 and CuRE are involved in nickel response [40].

Our fluorescence assays confirmed quantitatively that the activity of the *PrLmSAP* promoter is upregulated by many of the assayed stimulators (Table 1), especially by ABA, GA, cold, MeJA, PEG, NaCl, Cu, Cd, Zn, and Al, which are consistent with the identified *cis-acting* elements. Our results are also consistent with previous reports regarding the promoters of *OsMTP11* [65], OsM2Tb [66], *OsMT-I-4b* [67], *AtMRP3* [68], *AtPCS* [69] and *AtM2Tb* [70], which are metal-induced promoters, linking heavy metal-specific cellular responses in plants.

As mentioned before, the exact mode of action of SAPs proteins is still unknown even though elements involved in their signalling have started to emerge [71]. While research has been carried out into the various parameters that can induce SAPs (protein or transcript), there is a scarcity of information of how these varied treatments lead to the induction of SAPs at a molecular level. As promoters are the endpoint of signalling pathways, the study of their activation by stimulators serves to reveal the transcriptional response to treatments, whereas analysis of transcript abundance, protein, or activity levels also incorporate a variety of post-transcriptional regulatory mechanisms. Thus, the analysis of the promoter region of *SAPs* genes can provide a better understanding of their induction at a transcriptional level. Several works have studied the sequences of the regulatory region of different *SAP* genes (*AlSAP*, *OsSAP1*, *OsSAP6*, *OsSAP8*, and *OsSAP9*, *AtSAP13*) revealed the presence of various *cis*-acting regulatory elements involved in abiotic/biotic stress-responsive gene expression [26, 43, 44, 72, 73]. In this study, we compared in detail the promoter regions of *LmSAP* and other three genes encoding for A20/AN1 zinc-finger proteins from *Arabidopsis thaliana* (*PrAtSAP5* (*At3g12630*), *PrAtSAP13* (*At3G57480*)) and *Oryza sativa* (*PrOsSAP9* (*Os07g07350*.*1*). AtSAP5 [45] and OsSAP9 [44] proteins carry one A20 and one AN1 zinc-finger domains, while AtSAP13 [72] carries two AN1 zinc finger domains and an additional Cys2-His2 domain. These three proteins have probed roles in conferring tolerance to several abiotic stresses, including those studied by our group for LmSAP. Overexpression of *AtSAP5* in transgenic *Arabidopsis* confered tolerance to salinity and dehydration stresses [74]; overexpression of *OsSAP9* in tobacco plants afforded tolerance to high and low temperature and oxidative stress but led to an increased sensitivity to dehydration and salt stresses [44] while *AtSAP13* overexpression lines showed tolerance to toxic metals (AsIII, Cd, and Zn), drought, and salt stresses [72]. Transcription levels for these genes changed accordingly to these responses. Our *in silico* comparison of the promoter sequences revealed common elements but a consistently higher number of *cis*-regulatory elements for *PrLmSAP*. This is a very interesting finding, as it is well known that motif copy number correlated with promoter strength [75]. Thus, it will be worthy to establish whether *PrLmSAP* can induce higher transcription for other *SAP*- and stress-related genes.

We also compared the *cis*-regulatory elements of *PrLmSAP* with those present in the root-specific promoters *PrOsETHE1*, *PrOsNramp5*, *PrOsLsi1*, and *PrOsWOX11*, which are highly expressed in the roots in a tissue-specific manner. Interesting differences between *PrLmSAP* and the other promoters include the simultaneous presence of *cis*-elements involved in the signalling of the phytohormones ABA, GA, ethylene, and SA, as well as *cis*-elements with specific expression in the root primordia and root hairs [42].

Promoters and their contributing *cis*-elements intended for biotechnological applications should be functionally characterized in the same plant species targeted for genetic engineering. Our interest in characterizing *PrLmSAP* expression in rice was twofold: first, worldwide rice production is challenged by the concurrent biotic and abiotic stresses which reduce productivity and affect grain quality. In consequence, promoters for multiple stress responses, such as *PrLmSAP*, could support the development of more stress-tolerant rice varieties. Second, plant response to stresses in a target environment must be fine-tuned; in the last decade remarkable progress has been made regarding the comprehension of stress-signalling networks in plants however the practical exploitation of this knowledge for transgene expression in plants is limited, partly due to the shortage of promoters with the desired expression (developmental- and tissue-specific or condition dependent) that allow for the fine-regulation of the target genes expression, especially in monocot species [16, 42, 76]. The results presented in this study position *PrLmSAP* as a good candidate for directing the expression of target tolerance-genes in rice in a tissue- and condition-specific manner.

## Conclusions

We isolated and characterized the promoter *PrLmSAP* from *L*. *maritima* that control the expression of *LmSAP*, a gene encoding for an A20/AN1 zinc-finger protein. We demonstrated that a wide range of abiotic stresses induces *PrLmSAP* in a tissue-specific manner. We propose that *PrLmSAP* may be an effective promoter for controlling the expression of stress tolerance genes since it drives low, constitutive transgene expression under normal conditions but is highly inducible in response to abiotic stress. *PrLmSAP* activation by heavy metals positions it as a novel tool for regulating the expression of target genes conferring heavy metal tolerance to plants or for enhancing the accumulation of heavy metals in plants used in bioremediation. Finally, *PrLmSAP* inducibility by wounding is also a promising feature for genetic engineering of biotic stress-resistance in plants. Globally, our results improve the understanding of how *cis*-elements drive gene expression and favour the use of inducible promoters in plant genetic engineering.

## Supporting information

S1 Fig Graphical distribution of *cis*-regulatory elements identified by *in silico* comparison of *LmSAP* gene promoter with rice root-specific promoters. (TIF)

S2 Fig The histochemical localization of *β*-glucuronidase (GUS) activity in representative transgenic rice plants harboring L13-*PrLmSAP*::*gusA* during seed germination, in flowers and panicle. (**A–C**) Seedlings grown on MS medium at 12 h (**A**), 24 h (**B**) and 72 h (C). **(D)** Leaf, **(E)** Stem, **(F)** Lateral root, **(G)** Seminal root, (**H–I**) Reproductive organs of the transgenic plants, **(J)** Detection of GUS in L13-*PrLmSAP*::*gusA* transgenic rice leaves and stems following wounding. **(K)** Transversal vibratome section through a leaf blade of transgenic rice harboring the L13-*PrLmSAP*::*gusA*. (**L)** Transversal vibratome section through the seminal root of transgenic rice harboring the L13-*PrLmSAP*::*gusA*. Intense blue color indicates positive GUS signals. Bars 50 μm.
(TIF)

S3 Fig Induction by abiotic stress treatments of the *PrLmSAP* promoter in L13 transgenic rice. Histochemical assays on representative 7 DAG (days after germination) seedlings stressed with NaCl (150 mM), 10% PEG-6000, cold (4°C), GA (50 μM), ABA (100 μM), MeJA (100 μM), CuSO_4_ (100 μM), ZnSO_4_ (100 μM), CdCl_2_ (50 μM) or AlCl_3_ (50 μM) for 24 h.
(TIF)

S1 Table Comparison between putative *cis*-regulatory elements of *LmSAP* gene promoter and four root-specific promoters from rice. (DOC)

S1 Raw images (PDF)
