# Supplementary figures and images for: Characterization of a novel LmSAP gene promoter from Lobularia maritima: Tissue specificity and environmental stress responsiveness
Source: PLoS One. 2020 Jul 31;15(7):e0236943. doi: 10.1371/journal.pone.0236943 (PMC7394455; doi:10.1371/journal.pone.0236943)

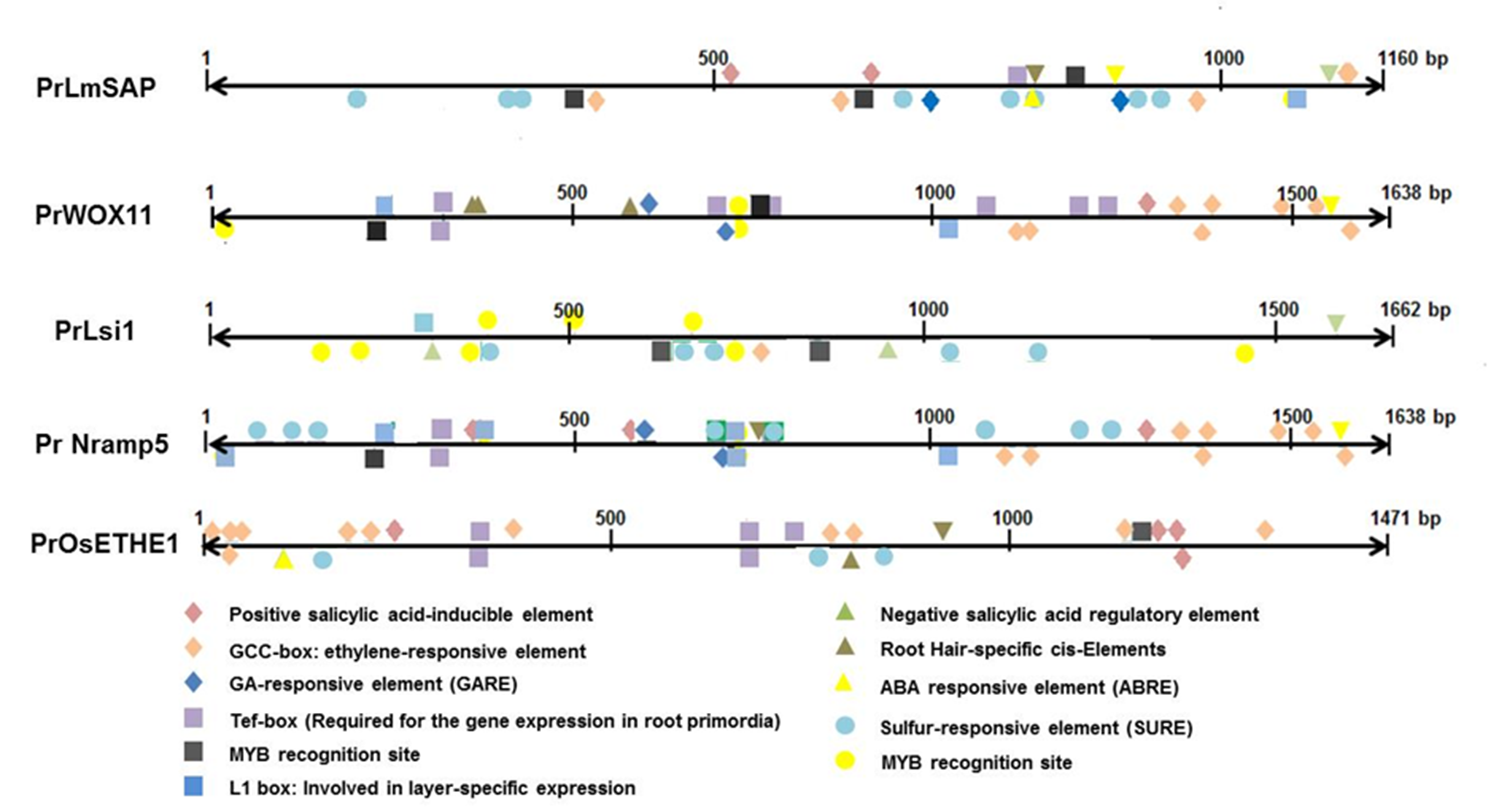

Supplement: S1 Fig — (TIF) [file pone.0236943.s001.tif]

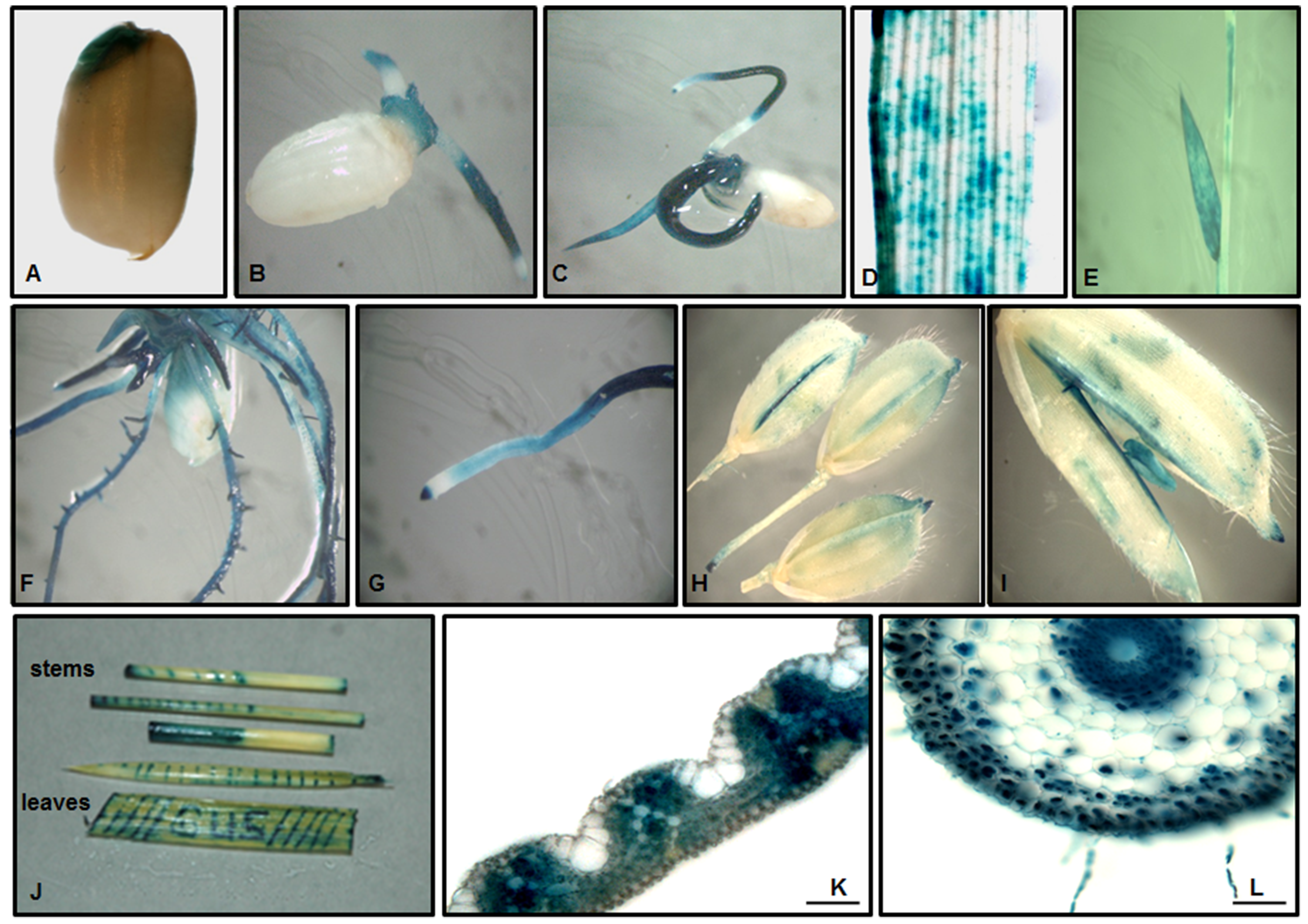

Supplement: S2 Fig — (A–C) Seedlings grown on MS medium at 12 h (A), 24 h (B) and 72 h (C). (D) Leaf, (E) Stem, (F) Lateral root, (G) Seminal root, (H–I) Reproductive organs of the transgenic plants, (J) Detection of GUS in L13-PrLmSAP::gusA transgenic rice leaves and stems following wounding. (K) Transversal vibratome section through a leaf blade of transgenic rice harboring the L13-PrLmSAP::gusA. (L) Transversal vibratome section through the seminal root of transgenic rice harboring the L13-PrLmSAP::gusA. Intense blue color indicates positive GUS signals. Bars 50 μm. (TIF) [file pone.0236943.s002.tif]

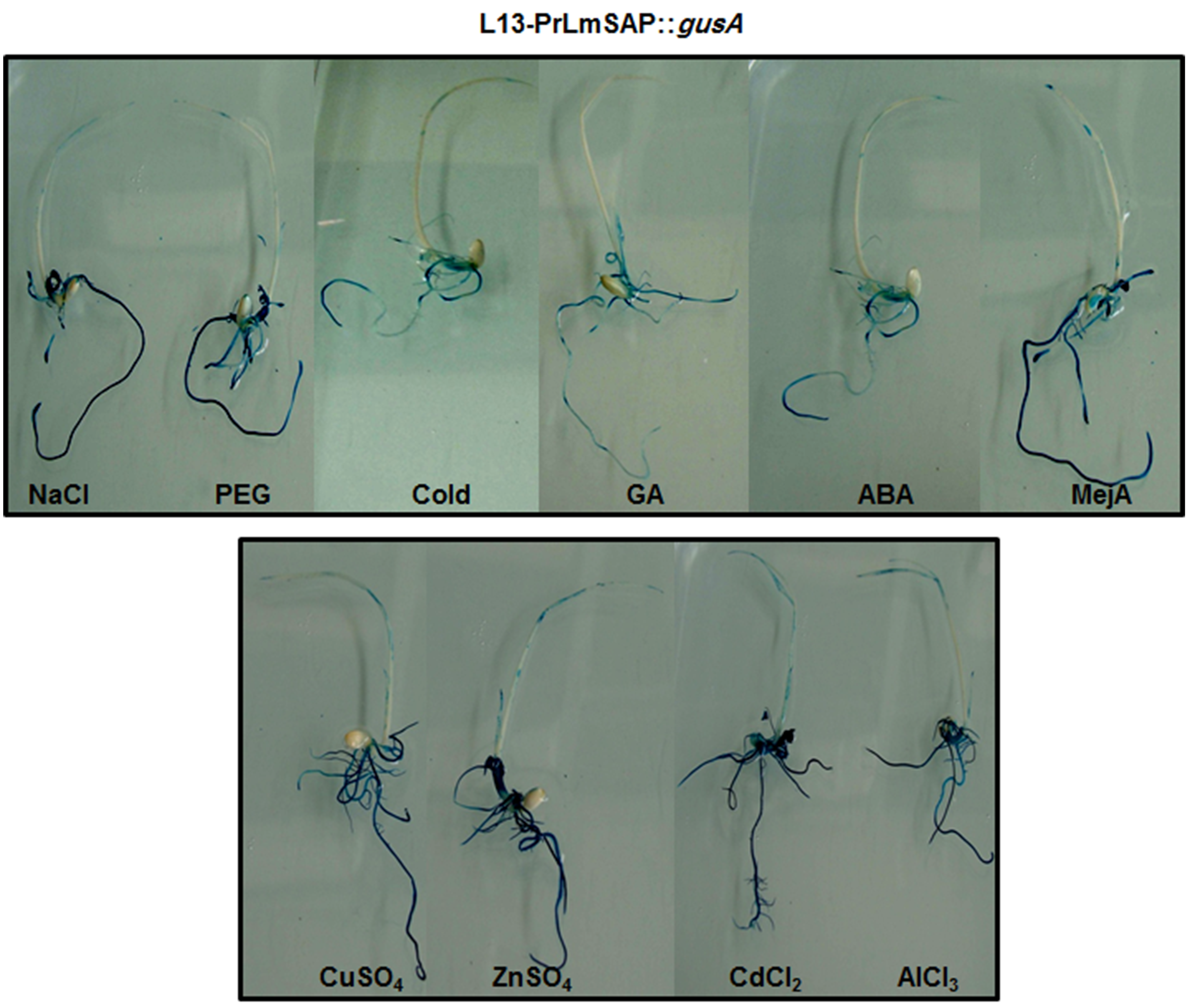

Supplement: S3 Fig — Histochemical assays on representative 7 DAG (days after germination) seedlings stressed with NaCl (150 mM), 10% PEG-6000, cold (4°C), GA (50 μM), ABA (100 μM), MeJA (100 μM), CuSO4 (100 μM), ZnSO4 (100 μM), CdCl2 (50 μM) or AlCl3 (50 μM) for 24 h. (TIF) [file pone.0236943.s003.tif]
