# Supplementary material for: Characterization of a novel LmSAP gene promoter from Lobularia maritima: Tissue specificity and environmental stress responsiveness
Source: PLoS One. 2020 Jul 31;15(7):e0236943. doi: 10.1371/journal.pone.0236943 (PMC7394455; doi:10.1371/journal.pone.0236943)
Supplement: S1 Table — (DOC) [file pone.0236943.s004.doc]

|  |  | **PrLmSAP** | | **PrOsETHE1**  **(LOC_Os01g47690)** | | **Pr Nramp5**  **(LOC_Os07g15370)** | | **PrLsi1**  **(LOC_Os02g51110)** | | **PrWOX11**  **(LOC_Os07g48560)** | |
| --- | --- | --- | --- | --- | --- | --- | --- | --- | --- | --- | --- |
| **Motif Name** | **Sequence** | **N° of position** | **Similar Score** | **N° of position** | **Similar Score** | **N° of position** | **Similar Score** | **N° of position** | **Similar Score** | **N° of position** | **Similar Score** |
| **Root Hair-specific**  **cis-Elements** | ACGTGC | 1 | 1 | 4 | 1 | 4 | 1 | *** | *** | *** | *** |
| **Tef-box (Required for the gene expression in root primordia)** | AGGGGCATAATGGTAA | 1 | 0.75 | 4 | 0.99 | 1 | 0.99 | *** | *** | 1 | 1 |
| **Positive salicylic acid-inducible element** | ACGTCATAGA | 2 | 0.7 | 5 | 0.7 | 1 | 0.7 | 7 | 0.7 | 4 | 0.7 |
| **Negative salicylic acid regulatory element** | TCTACGTCAC | 1 | 0.7 | *** | *** | 2 | 0.8 | 3 | 0.7 | *** | *** |
| **ABA responsive element (ABRE)** | ACGTG | 2 | 1 | 4 | 0.96 | 1 | 0.86 | *** | *** | 1 | 0.89 |
| **GA-responsive element (GARE)** | TAACAAR | 2 | 1 | *** | *** | 3 | 1 | *** | *** | *** | *** |
| **Sulfur-responsive element (SURE)** | GAGAC | 9 | 1 | 4 | 0.8 | 6 | 1 | 7 | 0.8 | 9 | 1 |
| **GCC-box**  **Ethylene-responsive element** | GCCGCC | 5 | 1 | 16 | 1 | *** | *** | 1 | 1 | 19 | 1 |
| **MYB recognition site** | WAACCA | 3 | 0.95 | 1 | 1 | 1 | 1 | 1 | 1 | *** | *** |
| **MYB recognition site** | YAACKG | 1 | 1 | 1 | 1 | 4 | 1 | 8 | 1 | *** | *** |

**S1 Table.** Comparison between putative *cis*-regulatory elements of *LmSAP* gene promoter and four root-specific promoters from rice.

(***): Motif unidentified
